# Mutations in the heat-shock protein A9 (*HSPA9*) gene cause the EVEN-PLUS syndrome of congenital malformations and skeletal dysplasia

**DOI:** 10.1038/srep17154

**Published:** 2015-11-24

**Authors:** Beryl Royer-Bertrand, Silvia Castillo-Taucher, Rodrigo Moreno-Salinas, Tae-Joon Cho, Jong-Hee Chae, Murim Choi, Ok-Hwa Kim, Esra Dikoglu, Belinda Campos-Xavier, Enrico Girardi, Giulio Superti-Furga, Luisa Bonafé, Carlo Rivolta, Sheila Unger, Andrea Superti-Furga

**Affiliations:** 1 Department of Medical Genetics, University of Lausanne, Lausanne, Switzerland; 2 Centre for Molecular Diseases, Department of Pediatrics, Lausanne University Hospital (CHUV), Lausanne, Switzerland; 3 Sección Genética, Hospital Clínico Universidad de Chile, and Sección Citogenética, Laboratorio, Clínica Alemana de Santiago, Santiago, Chile; 4 Unidad de Genética, Hospital Regional Rancagua, Rancagua, Chile; and ICBM, Facultad de Medicina, Universidad de Chile, Santiago, Chile; 5 Division of Pediatric Orthopaedics, Seoul National University Children’s Hospital, Seoul, Republic of Korea; 6 Department of Pediatrics, Pediatric Clinical Neuroscience Center, Seoul National University Children’s Hospital, Seoul, Republic of Korea; 7 Department of Biomedical Sciences, Seoul National University College of Medicine, Seoul, Republic of Korea; 8 Department of Radiology, Woorisoa Children’s Hospital, Saemalro, Guro-gu, Seoul 08291, Republic of Korea; 9 CeMM Research Center for Molecular Medicine of the Austrian Academy of Sciences, 1090 Vienna, Austria; 10 Medical Genetics Service, Lausanne University Hospital (CHUV) and University of Lausanne, Lausanne, Switzerland; 11 Department of Pediatrics and Pediatric Surgery, University of Lausanne and Lausanne University Hospital (CHUV), Lausanne, Switzerland

## Abstract

We and others have reported mutations in *LONP1*, a gene coding for a mitochondrial chaperone and protease, as the cause of the human CODAS (cerebral, ocular, dental, auricular and skeletal) syndrome (MIM 600373). Here, we delineate a similar but distinct condition that shares the epiphyseal, vertebral and ocular changes of CODAS but also included severe microtia, nasal hypoplasia, and other malformations, and for which we propose the name of EVEN-PLUS syndrome for epiphyseal, vertebral, ear, nose, plus associated findings. In three individuals from two families, no mutation in *LONP1* was found; instead, we found biallelic mutations in *HSPA9*, the gene that codes for mHSP70/mortalin, another highly conserved mitochondrial chaperone protein essential in mitochondrial protein import, folding, and degradation. The functional relationship between LONP1 and HSPA9 in mitochondrial protein chaperoning and the overlapping phenotypes of CODAS and EVEN-PLUS delineate a family of “mitochondrial chaperonopathies” and point to an unexplored role of mitochondrial chaperones in human embryonic morphogenesis.

Recently, we and others reported on the identification of mutations in *LONP1* as the cause of the human genetic disorder, CODAS (cerebral, ocular, dental, auricular and skeletal) syndrome (MIM 600373)^1^,^2^. *LONP1* codes for a phylogenetically conserved protein of the mitochondrial matrix that has both chaperone and protease activity. Subsequently, we have identified patients with a condition that shares the skeletal features of the CODAS syndrome but includes a distinct craniofacial dysmorphism and cardiac and intestinal malformations.

A review of the literature suggests that one sporadic patient reported as having CODAS^3^, two sibs reported as having an EVE (epiphyseal-vertebral-ear) syndrome^4^, and a further sporadic patient reported as having caudal regression with anal atresia and spondylo-epiphyseal dysplasia^5^ may have had the disorder we describe here, for which we propose the name of EVEN-PLUS syndrome for epiphyseal, vertebral, ear, nose, plus associated findings.

We report here the identification of recessive mutations in the *HSPA9* gene in our three patients with this syndrome. The *HSPA9* gene codes for a mitochondrial chaperone that has been implicated in different physiologic processes, and thus has been known under several different names (heat-shock 70 kda protein 9 (HSPA9, HSPA9B), mortalin and mortalin 2 (MOT/MOT2), 75 KDa glucose-regulated protein (GRP75), among others)^6^,^7^,^8^. Notably, the HSPA9 protein participates in, and is necessary for, the proteolytic activity of LONP1^9^,^10^,^11^,^12^.

## Clinical Reports

### Patient 1

**Figure 1:**
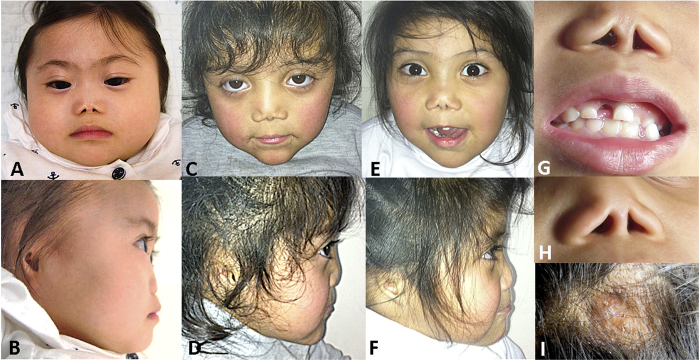
Photographs of patients 1 (A,B), 2 (C,D) and 3 (E,F). Common features include hypoplasia of the midface and of the nasal bones giving a flat nose, triangular nares, arched eyebrows with synophrys, and hypoplastic-dysplastic external ears. Panel G and H show the flat nose with triangular nares in patients 3 and 2, respectively, with a single central incisor in patient 3; and panel I shows the lesion of congenital aplasia cutis on the vertex of patient 3.

**Figure 2:**
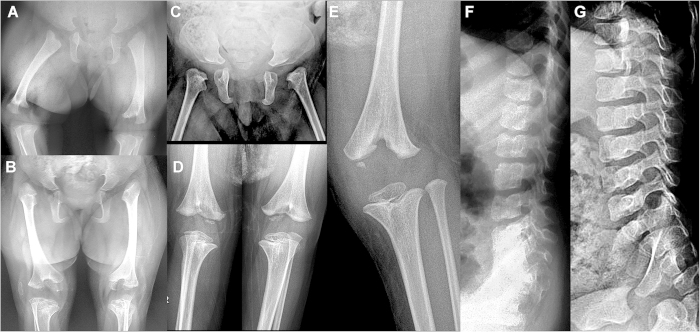
Skeletal-radiographic features. Panels A and B: patient 2 at birth (**A**) and at age 5 years (**B**) showing underossified pubic bones; bilateral dysplasia of the femoral heads at birth resulting in hip dislocation at age 5 yrs; and a “bifid” appearance of the distal femur with epiphyseal delay at birth, with dysplastic epiphyses that are “socketed” in the bifid femur at age 5 yrs; and small, laterally dislocated patellae. Panels C, D and E: Patient 1 at birth (**C**) with dysplastic femoral heads and at age 4 years (**D,E**) showing the bifid distal femur and the markedly dysplastic distal femoral epiphyses. The proximal tibial epiphyses are too small but less severely affected. Panels F and G: lateral lumbar spine of patient 3 at age 3 yrs (**F**) and of patient 1 at age 4 yrs (**G**) showing remnants of coronal clefts of the vertebral bodies. The clefts are prominent at birth and gradually disappear as ossification progresses.

This girl was the first child of a non-consanguineous couple of Korean origin. Upon specific questioning, there was no history of Parkinson disease in parents or grandparents. She was born at term (39 weeks) with a length of 38 cm and a weight of 2.2 kg. Her face showed midface hypoplasia with markedly hypoplastic nasal bones, giving her a flat nose with nares that were triangular in shape (Fig. 1). She had arched eyebrows and synophrys. Her external ears were markedly small and poorly formed (Fig. 1), while the ear canal was present. She was noted to have anal atresia that was subsequently surgically corrected. An atrioseptal defect (ASD) was present at birth but repeat ultrasound at age 20 months showed that it had closed spontaneously. At age 16 months, her developmental quotient was approximately 80; a cerebral MRI was normal, an abdominal ultrasound examination did not show abnormalities of kidneys or urinary tract, an ophthalmologic examination was normal (specifically, no cataracts were observed), and a CGH array study gave normal results. Skeletal radiographs obtained at age 16 months at 4 yrs showed lateral vertebral clefts, dysplasia of the proximal femurs and acetabula, “bifid” distal femurs and marked epiphyseal dysplasia at her knees (Fig. 2). These skeletal changes were considered typical of CODAS syndrome. However, no variants in the *LONP1* gene were found.

### Patient 2

This girl was born to a consanguineous couple of Chilean origin; the parents are uncle once removed and niece. Family history was negative for Parkinson disease. Short long bones were noted prenatally. She was born at 38 weeks’ gestation with a length of 39 cm (markedly below the 3^rd^ percentile), a weight of 2.8 kg, and a head circumference of 33,5 cm (slightly below the 10th percentile). Her karyotype was normal in blood cells. When seen in the genetics clinic at age 5 years, her weight was 12,5 kg, and she had severe short stature (markedly below the 3rd percentile for Chilean children) with a height of 80 cm; her head circumference 47,8 cm (below the 3^rd^ percentile). She had severe bilateral microtia with apparently normal external ear duct, arched eyebrows with mild synophris, and a very flat nose with nares that were triangular in shape (Fig. 1). Her cranial fontanels were still open, she had two lateral hair whorls and a small area of aplasia cutis on her cranium. Her limbs looked short. An echocardiography showed a small ASD. Cerebral, abdominal and renal ultrasound scans gave normal results. Her language development was appropriate and she attended kindergarten. Radiographic findings at birth included dysplasia of the femoral heads and of the acetabulum as well as “bifid” distal femurs (Fig. 2). At age 5 yrs, the proximal femoral epiphyses were not ossified, the femoral heads appeared to be dislocated, and the epiphyses at the knee were dysplastic.

### Patient 3

This girl is the younger sister of patient 2. Oligohydramnios and short long bones were noted prenatally. Born after 38 weeks of gestation, she was very short at birth, while weight and head circumference were at the lower limit of normal (weight, 2.7 kg; length, 39 cm; and head circumference, 34,5 cm); clinically, she appeared to have short limbs. She also had rectal atresia without fistula that required a surgical colostomy. She had a normal female karyotype in blood cells. At age 8 months, developmental delay was diagnosed. An echocardiography done at age 2 years 6 months showed persistent foramen ovale and aneurysmatic septum. When she was seen in the genetics clinics at age 3 years 6 months, her weight was 12 kg and her height 79 cm (both below the 3^rd^ percentile). She had brachycephaly, severe bilateral microtia (Fig. 1) with apparently normal ear canal, aplasia cutis on the skull vertex, a very flat nose with triangular nares, arched eyebrows with mild synophrys, high palate, hypodontia (Fig. 1), short neck, and imperforate anus. She was developmentally delayed and attended a specialized Teleton institution. Imaging studies showed right vesico-ureteral reflux with right kidney nephropathy. A brain CT showed dysgenesis of the corpus callosum. Skeletal-radiographic findings are shown in Fig. 2. Radiographic findings were similar to those seen in her sister and included vertebral coronal clefts (Fig. 2) and agenesis of the coccyx.

## Results

### Exome sequencing identifies low-frequency mutations in the *HSPA9* gene in individuals with the EVEN-PLUS syndrome

**Figure 3:**
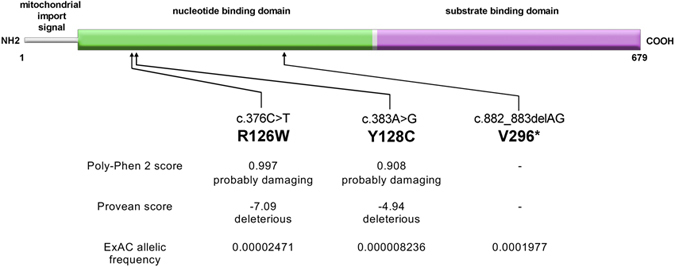
Scheme of the HSPA9 protein showing the localization of the mutations observed. The HSPA9 (mortalin) protein has a short mitochondrial import sequence and two main domains, the nucleotide (ATP/ADP) binding domain and the substrate binding domain (Dores-Silva *et al*., 2015). The two amino acid substitution affect the nucleotide binding domain; the truncation mutation predicts the loss of part of the nucleotide binding domain and all of the substrate binding domain (unless the mRNA undergoes nonsense-mediated decay; see Results). The lower part shows a summary of the pathogenicity prediction software (PolyPhen-2, http://genetics.bwh.harvard.edu/pph2/) and PROVEAN, http://provean.jcvi.org/index.php) as well as the allelic frequencies in the ExAC project (exac.broadinstitute.org/gene/ENSG00000113013).

The sequential analysis of the variants identified in the exomic sequence of the patients is presented in table S1. Variants were filtered for non-synonymy, for rarity, and for quality. Subsequently, genes were scored for the presence of either two variants at heterozygosity, or one or more variants at homozygosity, in the affected sibs as well as in the sporadic patient. There was only one gene that fit all criteria, namely, *HSPA9*. Patient 1 was found to be heterozygous for variants c.383A > G (p.Y128C) and c.882_883delAG (p.V296*). Patients 2 and 3 were found to be homozygous for variant c.376C > T (p.R126W). Both R126 and Y128 are extremely conserved (supplementary Figure S1). Results of prediction software PolyPhen-2^13^ and Provean^14^ suggested damaging results on protein structure (Fig. 3). The V296* truncation mutation abolishes more than half of the protein, including all of the substrate binding domain (Fig. 3); however, the premature termination codon is likely to promote nonsense-mediated decay. All mutations were confirmed by direct bidirectional Sanger sequencing of a second batch of genomic DNA; heterozygosity was confirmed in the unaffected parents. All three mutations were present at extremely low frequency in the ExAC browser (exac.broadinstitute.org/gene/ENSG00000113013) and were absent from the Exome Variant Server (http://evs.gs.washington.edu/EVS/) (Fig. 3). These frequencies are compatible with recessive inheritance of a rare disorder. Interestingly, the R126W mutation had been observed at heterozygosity in one individual out of three cohorts comprising over 1500 adult patients with Parkinson disease, and databases are since annotated with a possible role of this mutation in Parkinson disease^15^,^16^ (but see discussion below).

### 3D molecular modeling of HSPA9 and mapping of the affected amino acid residues

**Figure 4:**
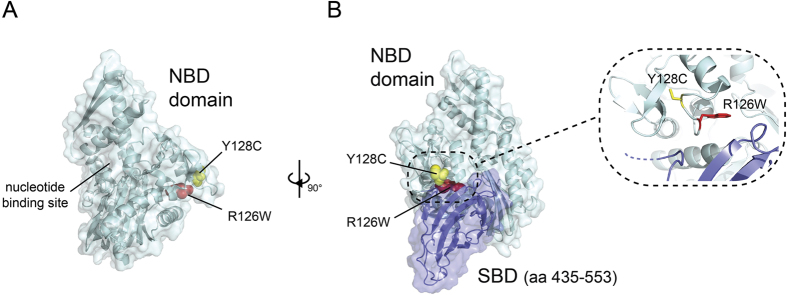
Localization of the mutations observed on the HSPA9 protein structure. (**A**) Mapping of the R126W and Y128C mutation of the crystal structure of the NBD (light blue). Both mutations lie on the surface of the protein, opposite to the nucleotide binding site. R126W is shown in red, Y128C in yellow. (**B**) Localization of the mutations in relation to the NBD/SBD interface (NBD and SBD shown in light green and purple respectively) in a model of the full length protein. Only a portion of the SBD is shown for clarity. Both mutations are located on a single loop near the interface, as shown in detail in the inset.

Mapping of the mutated amino acids on the available HSPA9 nucleotide binding domain structure (NBD)^17^ revealed that both R126W and Y128C are located next to each other on the surface of the protein, at some distance from the ATP/ADP binding site (Fig. 4A). Moreover, in our model the two mutations lie on a loop close to the predicted interface between the NBD and the substrate binding domain (SBD, Fig. 4B).

## Discussion

### Clinical delineation of the EVEN-PLUS syndrome

The identification of *LONP1* mutations in the CODAS syndrome allowed for the recognition of its wide phenotypic spectrum^1^,^2^. Subsequently, gene-based phenotypic sorting allowed us to identify a CODAS-related phenotype that is not associated with LONP1 mutations. This syndrome shares the skeletal features of the CODAS syndrome (vertebral and epiphyseal changes as shown in Fig. 2) but is further characterized by prenatal-onset short stature, a distinct craniofacial phenotype with microtia, a flat facial profile with flat nose and triangular nares, cardiac malformations, and other findings such as anal atresia, hypodontia, aplasia cutis, and others (see Fig. 1 and Table 1). Examples of this syndrome seem to have been previously reported: in 1990, Kozlowski *et al*. described a six-year-old boy, with « caudal regression and spondylo-epiphyseal dysplasia »^5^. The flat nose, dysplastic ears, and the combination of rectal and bladder incontinence with sacral agenesis observed in that boy are reminiscent those in our patients; the radiographic changes, namely, vertebral clefts, severe epiphyseal dysplasia, “bifid” appearance of the distal femur, and coccygeal agenesis, are virtually identical (see Fig. 2). In 2009, Marlin *et al*. reported on a boy diagnosed as having CODAS^3^; however, while the facial features in the CODAS syndrome are non-specific, the craniofacial features and the skeletal findings of that patient closely match those observed in our patients. Finally, Amiel and coworkers had reported, in 1994, two sisters who had epiphyseal and vertebral dysplasia in combination with dysplastic external ears; they discussed CODAS as a possible diagnosis but concluded that those sisters probably represented a separate disorder for which they coined the name of EVE (epiphyses, vertebrae, ear) syndrome^4^. Although no DNA could be obtained from these individuals to test for the presence of HSPA9 mutations, the clinical resemblance and similarity of radiographic features strongly suggest that these four patients had the same disorder as the one we describe here. Thus, we propose to retain the name suggested by Amiel and coworkers and to change it to “EVEN-PLUS syndrome” (for epiphyses, vertebrae, ears and nose, plus associated findings), reflecting the main clinical findings of the syndrome. While the skeletal findings in EVEN-PLUS are shared with CODAS, the facial features and the presence of associated malformations are distinct to the EVEN-PLUS syndrome.

### Recessive mutations in the HSPA9 gene are the basis of the EVEN-PLUS syndrome

Several lines of evidence support the causative role of the *HSPA9* mutations in the pathogenesis of this complex malformation syndrome. The genetic evidence consists in the rarity or absence of the variants in our in-house population (to exclude systemic technical errors) and in publicly available databases (ExAC, EVS); their presence at compound heterozygosity in patient 1 (non-consanguineous parents) as well as in homozygosity in patients 2 and 3 (consanguineous parents); the phylogenetic conservation of the affected residues (supplementary Figure 1); as well as the consequences at the protein level both for the premature stop codon mutation predicting the loss of more than half of the protein, and for the amino acid substitutions predicted *in silico* to be damaging (Fig. 3). The R126W and Y128C mutations are located on the NDB but are unlikely to directly affect this activity, due to their distance from the active site. However, the proximity of the mutation sites to the predicted NBD/SBD interface could result in the disruption of this interaction, thereby affecting the function of the protein. Interestingly, our 3D model of HSPA9/mortalin is consistent with a recent in solution analysis of the HSPA9 structure^6^, which places the NBD/SBD interface in similar orientation as the one predicted in our own molecular model. It seems therefore likely that the R126W and Y128C mutations result in similar consequences as the ones expected from the V296* mutation, which, if the mRNA escapes nonsense-mediated decay, abolishes the NBD/SBD interface and the whole substrate binding domain. Finally, albeit in another context, the R126W substitution has been shown to have adverse effects on HSPA9 protein function (see below).

### HSPA9/mortalin, a protein with (too) many functions?

The product of the HSPA9 gene, a 70 kDa heat-shock protein, has been extensively studied in the past 25 years and yielded precious insights on mitochondrial protein import and folding^6^,^7^,^8^,^10^. It is associated principally with the mitochondrial matrix but can be found in the cytoplasm as well as in the nucleus. Its main role is that of a chaperone that participates in the import of proteins from the cytosol to the mitochondrial matrix as well as their folding^18^,^19^. As a heat-shock protein with ATPase activity, it prevents the accumulation of unfolded, dysfunctional proteins^20^. Because of its different localizations and multiple binding partners, the HSPA9 protein has been known under several different names (mHSP70, mortalin, mot-2, GRP75) and has been associated with a large number of different processes ranging senescence to immortalization, oncogenesis, neurodegeneration, protection from oxidative stress, hematopoiesis, and even viral replication, among others^6^,^7^. Some of these studies, done in different contexts, have produced results that are relevant to our results. In 2005, Craven *et al*. studied a zebrafish mutant, *crimsonless*, ascertained for ineffective hematopoiesis. They identified a single amino acid substitution, G492E, in the *HSPA9b* gene (the zebrafish homologue of human *HSPA9*) as responsible for this phenotype. A morpholino knock-down model of *HSPA9b* effectively reproduced the *crimsonless* phenotype^21^. Although the study was focused on hematopoiesis, it was noted that the head and eyes appeared stunted as early as 38 hpf^21^; moreover, after 48 hpf all development appeared to halt, including further maturation of the musculature, fins, and internal organs, and the animals died around 72 hpf^21^, indicating that HSPA9b knockdown had more widespread effects on embryonic development. Other investigators have investigated a possible role of HSPA9 in the pathogenesis of Parkinson disease, based on the physical and functional relationship of HSPA9 (often referred to as *mortalin* in that literature) with parkins. In two studies on the possible role of genetic variations in *HSPA9* in the pathogenesis of Parkinson disease, the R126W mutation has been identified at heterozygosity in 1 of 330 Spanish patients^16^, and subsequently in 0 (none) of two cohorts of 286 and 1008 German patients^15^. In spite of the questionable statistical significance of this observation, expression studies using wild type and mutated HSPA9 were done, showing that the R126W mutation does impact the function of the HSPA9 protein measured by its effects on mitochondrial morphology, mitochondrial membrane potential, the production of reactive oxygen species^15^,^22^ and the cell sensitivity to exogenous oxidative stress^22^. These expression studies confirm that the R126W mutation is not a neutral polymorphism but does impair HSPA9 function, in accordance with *in silico* predictions and 3D mapping data.

### LONP1 mutations in the CODAS syndrome and HSPA9 mutations in the EVEN-PLUS syndrome suggest the existence of a family of “mitochondrial chaperonopathies”

The concept of families of phenotypes coming from genes in the same pathway, or related by a common function (such as the cohesinopathies, or the cholesterol biosynthesis defects, or the mucopolysaccharidoses) is well established. While the identification of mutations in a mitochondrial matrix protease (*LONP1*) in patients with the CODAS syndrome was unexpected^1^,^2^, the identification of mutations in a related gene (HSPA9) in EVEN-PLUS syndrome confirms the previous studies and suggests a common pathogenesis of the two syndromes. In fact, the functional relationship between HSPA9 and LONP1 in the mitochondrial chaperone-protease network^9^,^10^,^23^ and the phenotypic overlap between CODAS and EVEN-PLUS syndromes delineate a new family of disorders – the “mitochondrial chaperonopathies”.

### Mechanistic pathogenesis of the EVEN-PLUS and CODAS syndrome remains unclear but is likely to be different than that of classic mitochondriopathies

In spite of the numerous studies on HSPA9 function, and of the studies of Strauss *et al*. on *LONP1* mutations found in the CODAS syndrome^1^, the pathogenesis of the developmental defects associated with *LONP1* and *HSPA9* mutations remain unexplained. The phenotype of the CODAS syndrome, and even more so that of EVEN-PLUS, do not resemble those of other multisystem mitochondrial diseases; there is no indication of the “energy failure” process that is central to the pathogenesis of prototypical mitochondrial disorders such as the Pearson, Kerns-Sayre, MELAS, MERFF, or Alpers syndromes (reviewed by Andreux *et al*., 2013^24^). Recessive mutations in a different mitochondrial chaperone-protease, CLPP, have been identified as one cause of the Perrault syndrome of ovarian failure and hearing loss, a condition that more closely resembles the classic mitochondriopathies^25^. Many of the clinical features of EVEN-PLUS syndrome (microtia, small nose, abnormal hair patterns, anal atresia, sacral agenesis) are the consequence of disturbed embryonic morphogenesis. But how can HSPA9 malfunction result in dysmorphogenesis? Heat-shock genes are widely expressed in vertebrate development even in absence of stress factors, suggesting a role in development^26^. Specifically, HSPA9 is known to interact with FGF1^27^ as well as with Smad2, decreasing TGF-b signal transduction and affecting epithelial-mesenchymal transition^28^. Studies of the possible roles of HSPA9 in embryonic development may give an explanation for the defects observed in the EVEN-PLUS syndrome.

Awareness about the EVEN-PLUS syndrome and availability of molecular diagnosis will allow to recognize further cases, to increase our knowledge on the phenotypic spectrum associated with mutations in *HSPA9*, and to learn about the long-term outcome of affected individuals. The wealth on publications on the multiple roles of HSPA9/mortalin/mHSP70 notwithstanding, only these clinical observations may give us *in vivo* indications on the essential and non-essential roles of HSPA9 in prenatal development as well as over the entire human life-span.

## Methods

The study was done in accordance with regulations for studies on human subjects of the hospitals in Lausanne, Seoul, and Rancagua. In addition, approvals from the IRB of the Seoul National Hospital, and of the Ethics Commission of the Lausanne University Hospital, were obtained. Peripheral blood was obtained with informed consent from the patients and their parents and genomic DNA was extracted by routine methods. Fragmented genomic DNA was purified with AMPure XP beads and the quality of the fragmented DNA was assessed with an Agilent Bioanalyzer. Preparation of the exome enriched, barcoded sequencing libraries was perfomed using Agilent SureSelect Human All Exon v4 kit. The final libraries were quantified with a Qubit Fluorometer (Life Technologies) and the correct size distribution was validated with an Agilent Bioanalyzer. Libraries were sequenced on Illumina HiSeq 2000, generating 100 bp paired-end reads. Raw reads were aligned onto the hg19 reference genome with Novoalign (http://www.novocraft.com) and the data cleanup and variant calling were performed according to GATK Best Practices recommendations^29^. Variant filtering was made with Annovar^30^ and with own *perl* and *bash* scripts (available on request). Variants identified by this procedure were verified by direct PCR amplification of target exons from genomic DNA and bidirectional Sanger sequencing. A molecular model for the full-length human HSPA9 protein was generated with I-TASSER^31^. Figures were generated in the PyMOL Molecular Graphics System, Version 1.7.4 (Schrödinger, LLC).

## Additional Information

**How to cite this article**: Royer-Bertrand, B. *et al*. Mutations in the heat-shock protein A9 (*HSPA9*) gene cause the EVEN-PLUS syndrome of congenital malformations and skeletal dysplasia. *Sci. Rep*. **5**, 17154; doi: 10.1038/srep17154 (2015).

## Supplementary Material

Supplementary Information

## Figures and Tables

**Table 1:** Synopsis of clinical features in the three individuals with EVEN-PLUS syndrome and *HSPA9* mutations.

	Pat. 1	Pat. 2	Pat. 3
birth measurements	length 38 cm, weight 2.2 kg (at week 39); both values markedly below the normal range	length 39 cm, weight 2.8 kg (at week 38); length markedly below the normal range	length 39 cm, weight 2750 g (at week 38); length markedly below the normal range
nose	hypoplastic nose with vertical groove on tip (bifid tip) and triangular nares	hypoplastic nose with vertical groove on tip (bifid tip) and triangular nares	hypoplastic nose with vertical groove on tip (bifid tip) and triangular nares
ears	absent external ears (anotia), open ear duct	severe microtia with absent upper helix	absent external ears with open ear duct; possible hypoacusis
eyes	synophrys; no cataract	synophrys; no cataract	synophrys; no cataract
teeth			single upper central incisor, absence of some lateral incisors
skin	atopic dermatitis, sparse hair	two lateral hair whorls and area of aplasia cutis on the skull vertex	area of aplasia cutis on the skull vertex
heart	ASD (spontaneously closed at age 20 mos)	ASD (ostium secundum)	patent foramen ovale and aneurysmatic septum
gastrointestinal	anal atresia	normal abdominal ultrasonography	anal atresia
kidney/urogenital	No abnormalities on ultrasound	1 UTI at 1 year but normal renal ultrasonography	vesicoureteral reflux, hypoplastic right kidney
brain	normal MRI at age 5 mos	normal cerebral ultrasonography	agenesis of the corpus callosum with separated frontal horns
psychomotor development	Borderline-normal	Normal evaluation at kindergarten level, including language	Moderate developmental delay
*HSPA9 mutations*	p.Y128C/p.V296*	*pR126W/p.R126W*	*pR126W/p.R126W*
