# Supplementary material for: Mutations in the heat-shock protein A9 (HSPA9) gene cause the EVEN-PLUS syndrome of congenital malformations and skeletal dysplasia
Source: Sci Rep. 2015 Nov 24;5:17154. doi: 10.1038/srep17154 (PMC4657157; doi:10.1038/srep17154)
Supplement: Supplementary Information [file srep17154-s1.pdf]

SUPPLEMENTARY MATERIAL

Mutations in the heat-shock protein A9 (*HSPA9*) gene cause the EVEN-PLUS syndrome of congenital malformations and skeletal dysplasia

Beryl Royer-Bertrand[1,2], Silvia Castillo-Taucher[3], Rodrigo Moreno-Salinas[4], Tae-Joon Cho[5], Jong-Hee Chae[6], Murim Choi[7], Ok Hwa Kim[8], Esra Dikoglu[2], Belinda Campos-Xavier[2], Enrico Girardi[9], Giulio Superti-Furga[9], Luisa Bonafé[2], Carlo Rivolta[1], Sheila Unger[10], and Andrea Superti-Furga[2,11, *]

Supplementary Figure S1. Conservation of the two HSPA9 residues (R126 and Y128) affected by mutations in the EVEN-PLUS syndrome.

NP_004125.3	*H.sapiens*	HSPA9	679 aa	TKRLIGRRYDDPEVQKDIKNVPFKIV
XP_001171426.2	*P.troglodytes*	HSPA9	752 aa	TKRLIGRRYDDPEVQKDIKNVPFKIV
XP_001113234.1	*M.mulatta*	HSPA9	679 aa	TKRLIGRRYDDPEVQKDIKNVPFKIV
XP_531923.2	*C.lupus*	HSPA9	679 aa	TKRLIGRRYDDPEVQKDIKNVPFKIV
NP_001029696.1	*B.taurus*	HSPA9	679 aa	TKRLIGRRYDDPEVQKDIKNVPFKIV
NP_034611.2	*M.musculus*	Hspa9	679 aa	TKRLIGRRYDDPEVQKDTKNVPFKIV
NP_001094128.2	*R.norvegicus*	Hspa9	679 aa	TKRLIGRRYDDPEVQKDTKNVPFKIV
NP_001006147.1	*G.gallus*	HSPA9	675 aa	TKRLIGRRFDDSEVKKDIKNVPFKIV
NP_001001229.1	*X.tropicalis*	hspa9	670 aa	TKRLIGRRFDDAEVQKDLKNVPYKIV
NP_958483.2	*D.rerio*	hspa9	682 aa	TKRLIGRRFDDPEVKKDITNLSYKVV
NP_523741.2	*D.melanogaster*	Hsc70-5	686 aa	TKRLIGRRFDDAEIKKDLANLSYKVV
XP_309825.4	*A.gambiae*	AgaP_AGAP010876	641 aa	TKRLIGRRFEDPEVQKDLKVVPYKIV
NP_504291.1	*C.elegans*	hsp-6	657 aa	TKRLIGRRYEDAEVQRDIKQVPYKIV
NP_012579.1	*S.cerevisiae*	SSC1	654 aa	TKRLIGRRFEDAEVQRDIKQVPYKIV
XP_454960.1	*K.lactis*	KLLA0E22309g	641 aa	TKRLIGRRFEDAEVQRDIKQVPYKIV
NP_985899.2	*E.gossypii*	AGOS_AFR352C	642 aa	TKRLIGRRFKEPEVQRDIKEVPYKIV
NP_593459.1	*S.pombe*	ssc1	674 aa	TKRLIGRKFKDAEVQRDIKEVPYKIV
XP_003719632.1	*M.oryzae*	MGG_04191	669 aa	TKRLIGRKFTDPEVQRDIKEVPYKIV
XP_961753.2	*N.crassa*	NCU08693	668 aa	SKRLIGRRFDDPQTQKEMKMVPYKIV
NP_195504.2	*A.thaliana*	mtHsc70-1	682 aa	SKRLIGRTFDDPQTQKEMKMVPYKIV
NP_001175918.1	*O.sativa*	Os09g0491772	684 aa	TKRLIGRRFDDPEVQKDIKNVPFKIV

	Pat. 1	Pat. 2	Pat. 3
Number of exonic and splicing variants	23563	23930	23964
Number of non-synonymous variants	11698	11739	11689
Number of rare variants (<1%)	1276	813	793
Number of rare variants after quality control (QC)	668	458	465
Number of genes with two heterozygous variants	31	23	27
+ correctly segregating		*7*	
Number of genes with homozygous variants	12	18	22
+ correctly segregating		*12*	
Genes with variants shared by the three patients	1 (*HSPA9*)		

Table S1. Overview of the filtering of exonic and splicing variants observed in the patients.

Values refer to number of variants unless specified otherwise. Rare (<1%): Variant frequency = 1% or less in public databases – ExAC, ESP, and Wellderly, 1KG (from Complete Genomics). Rare (QC): Rare variants after quality control, i.e. after removal of (1) WES data of poor quality, with less than 20 reads per nucleotide and genotype quality less than 70, and (2) variants present in control WES processed by the same pipeline (to remove technical error). *Correctly segregating:* present in both affected sisters.
